# Role of Optical Coherence Tomography to Measure Retinal Nerve Fiber Layer Thickness in Patient With Chronic Obstructive Pulmonary Disease

**DOI:** 10.7759/cureus.25517

**Published:** 2022-05-31

**Authors:** Vishal Wagh, Pravin K Tidake, Gaurang Aurangabadkar

**Affiliations:** 1 Ophthalmology, Jawaharlal Nehru Medical College, Wardha, IND; 2 Respiratory Medicine, Datta Meghe Institute of Medical Sciences, Wardha, IND

**Keywords:** retinal nerve fiber layer, macular thickness, subfoveal choroidal thickness, spectral domain optical coherence tomography, chronic obstructive pulmonary disease

## Abstract

Aim: This study aimed to measure retinal nerve fiber layer thickness with spectral domain optical coherence tomography in patients diagnosed with stable chronic obstructive pulmonary disease. And, comparing the RNFL thickness of stable patients with mild to moderate COPD and severe to very severe COPD patients

Patients and methods: In this prospective case-control study, a total of 120 patients with chronic obstructive pulmonary disease (COPD) have been selected for measurement of retinal nerve fiber layer on spectral domain-optical coherence tomography (SD-OCT). This study included 120 eyes of participants among which 80 were COPD patients (G1) and 40 were healthy participants (control group: G2). The G1 group is further sub-classified into G1A included 40 eyes of 40 patients with mild to moderate COPD and G1B included 40 eyes of 40 patients with severe to very severe COPD. To determine the hypoxic status of patient arterial blood gas analysis was done. The subfoveal choroidal thickness and retinal nerve fiber thickness were calculated using spectral domain OCT, and these readings of OCT are compared with COPD.

Results: There was no statistically significant difference between groups according to demographic data. Mean peripapillary retinal nerve fiber layer (RNFL) thickness on SD-OCT was statistically significantly thinner in mild to moderate COPD (106.76±4.39) group and severe to very severe COPD (100.75±4.66) group than in the control group (108.51±4.39); this thinning was more pronounced with increase in the severity of COPD. On comparing macular thickness between three groups, it was found that there were no statistically significant differences in macular thickness (MT) (p=0.684)*. *Subfoveal choroidal thickness (SFCT) was significantly thinner in the COPD groups compared to the control group.

Conclusion: Changes in the SD-OCT and retinal nerve fiber layer (RNFL) thickness could be used as indicators for the severity of COPD. And, it can also aid in the early intervention to hypoxic changes in the retina there by decreasing the chances of visual field defect

## Introduction

A chronic obstructive pulmonary disease (COPD) diagnosis is characterized by low-grade systemic inflammation causing a variety of systemic symptoms and sign [1,2]. In COPD, inhaled irritants, such as cigarettes, are the primary cause of inflammation. By causing the release of leukocytes and thrombocytes from the bone marrow and activating circulating leukocytes and endothelial cells, these inhaled irritants trigger the production of inflammatory mediators such as c-reactive protein (CRP), interleukin, and fibrinogen and initiate inflammatory events [3]. Additionally, the inflammatory mediators cause tissue damage and maintain the inflammatory process. COPD patients can also experience eye complications. It has been found that chronic pulmonary diseases like COPD, asthma, lung cancer, and pulmonary fibrosis are associated with low corneal endothelial cell density preoperatively [4]. According to Soler et al., COPD patients have lower endothelial function reserve [5]. Another study has found a relationship between cataracts and COPD exacerbations [6]. A number of systemic disorders involve the retinal and choroidal microvasculature [7]. Several fine ocular structures are thought to be involved in hypoxia and systemic inflammation, including the choroid, macula, retinal nerve fiber layer, and retinal vascular system. Spectral domain-optical coherence tomography (SD-OCT) can be used to assess all of them quantitatively and qualitatively [8].

## Materials and methods

This was a prospective case-control study approved by the institutional ethics committee of DMIMSU. Written consent of all patients was taken. And the study was adherent to the principles of the declaration of Helsinki. The study was conducted at the ophthalmology and chest medicine department of Jawaharlal Nehru Medical College for a period of one year.

This study included 120 eyes of participants among which 80 were COPD patients (G1) and 40 were healthy participants (control group: G2). According to Global Initiative for Chronic Obstructive Lung Disease (GOLD) criteria, the G1 group is further subclassified into G1A included 40 eyes of 40 patients with mild to moderate COPD and G1B included 40 eyes of 40 patients with severe to very severe COPD.

Inclusion and exclusion criteria

Patients with COPD having pulse oximetry measurement <94%, age more than 40 years, visual acuity of 6/36 or better, spherical refractive error less than 3D, astigmatic refractive error less than 3D, intraocular pressure below 21 mmHg were included.

Patients with recent ocular surgery (within one month); age <40years; patients with hypertension, diabetes mellitus (DM), cardiac failure; patients with age-related macular degeneration and glaucoma were excluded. A comprehensive ophthalmic examination, including best corrected visual acuity, slit-lamp examination, intraocular pressure (IOP) measurement, and SD-OCT, was performed on all participants.

Examination of COPD patients

All patients were subjected to a thorough physical examination. Following investigations were performed on each individual patient.

Spirometry

The patients were subjected to a pulmonary function test to stage the severity of COPD according to Global Initiative for Chronic Obstructive Lung Disease (GOLD) criteria.

Pulmonary function test: A pulmonary function test was performed using an RMS Helios 401 spirometer in compliance with American Thoracic Society/European Respiratory Society (ATS/ERS) standards. The test showed the following results: FEV1, FVC, FEV1/FVC ratio, SVC, and MVV. The main parameters utilized to stage COPD patients according to Global Initiative for Chronic Obstructive Lung Disease (GOLD) were FEV1, FVC, and the FEV1/FVC ratio (Table 1). Spirometry was performed post-bronchodilator in all patients to rule out bronchial asthma (metered-dose inhaler {MDI} salbutamol 400 mcg with spacer).

**Table 1:** Global Initiative for Chronic Obstructive Lung Disease (GOLD) criteria COPD: chronic obstructive pulmonary disease

GOLD stage	COPD severity	FEV­_1 _(% predicted)
GOLD 1	Mild	≥ 80% predicted
GOLD 2	Moderate	50-79% predicted
GOLD 3	Severe	30-49% predicted
GOLD 4	Very severe	< 30% predicted

Optical Coherence Tomography Measurements

An experienced technician performed the OCT measurements on a single patient at the same visit using tropicamide 0.8% (w/v) and phenylephrine 5% (w/v) using the Cirrus HD 500 spectral OCT platform (Dublin, CA: Carl Zeiss Meditec). We retained only high-quality images (signal strength ≥ 7) that clearly showed the peripapillary and macular regions. A 6 × 6 mm^2^ area centered on the optic disc was used to measure the peripapillary retinal nerve fiber layer (RNFL) thickness using an optic disc cube scan protocol (200 × 200 pixels). The average RNFL thickness (and those of the four quadrants; superior, nasal, inferior, and temporal) was automatically calculated and reported.

## Results

Demographic and clinical characteristics of the study population

The current study included 120 eyes of participants among which 80 COPD patients (G1) and 40 healthy participants as a control group (G2). The G1 group is further sub-classified into G1A included 40 eyes of 40 patients with mild to moderate COPD and G1B included 40 eyes of 40 patients with severe to very severe COPD. There was no statistically significant difference between groups according to the demographic data (p >0.05). IOP was statistically significantly different between groups, and it was higher in the G1B group with a mean IOP of 14.33 mmHg followed by G1A and G2 (Table 2).

**Table 2:** Demographic and clinical characteristics of the study population *P-value >0.05 NS. **P-value <0.05 S. NS: non-significant; S: significant

Parameter		Group 1A	Group 1B	Group 2	F/χ2*	p-Value
		Mild to moderate COPD	Severe to very severe COPD	Control		
Age (years)		56.20±4.22	57.88±4.83	57.28±4.46	1.381	0.255
Sex	Female	12 (30%)	15 (37.5%)	9 (22.5%)	0.752	0.686
	Male	28 (70%)	25 (62.5%)	31 (77.5%)		
IOP (mmHg)		13.20±2.51	14.33±4.06	12.15±2.26	4.960*	0.009**

Pulmonary measurements of the COPD groups

In comparing the arterial blood gas (ABG) between two groups (G1A and G1B) with COPD. Majority of the patients with types 1 and 2 respiratory failure are seen in G1B with 25% and 22.5% compared to G1A with 12.5% and 7.5%. A statistically significant difference was observed between the two groups (p >0.001). The oxygen saturations of the patients in the G1B with severe COPD were significantly lower than the group with G1A (p >0.001) (Table 3).

**Table 3:** Comparison between COPD groups according to ABG and spirometry *P-value >0.05 NS. **P-value <0.001 HS. FEV1: forced expiratory volume in the first second; FVC: forced vital capacity; NS: non-significant; HS: highly significant; ABG: arterial blood gas; COPD: chronic obstructive pulmonary disease; RF: respiratory failure

Parameters		Group 1A		Group 1B		Independent sample t-test*	p-Value
		Mild to moderate COPD		Severe to very severe COPD			
ABG		Frequency	%	Frequency	%	14.417	<0.001**
Normal		32	80%	21	52.5%		
Type 1 RF		5	12.5%	10	25		
Type 2 RF		3	7.5%	9	22.5%		
		Mean	SD	Mean	SD		
O_2_ saturation		95.70	±2.19	92.50	±4.30	4.144	<0.001**
Spirometry	% FEV1	69.95	±10.88	34.63	±11.27	14.083	<0.001**
	% FVC	81.63	±14.80	51.70	±10.90	10.167	<0.001**
	FEV1/FVC	0.62	±0.06	0.55	±0.11	3.467	<0.001**

OCT measurements of the study populations

SD-OCT was done in all 120 patients in G1A (mild to moderate COPD), G1B (severe to very severe COPD), and G2 (control group). In all four groups, subfoveal choroidal thickness, macular thickness, and an average peripapillary RNFL thickness are measured. Peripapillary RNFL thickness is measured in all four quadrants (superior, nasal, inferior, and temporal quadrant) (Table 4).

**Table 4:** Comparison between all groups regarding subfoveal choroidal thickness, macular thickness, and peripapillary RNFL thickness *P-value >0.05 NS. **P-value <0.001 HS. ***P-value <0.05 S. S: significant; NS: non-significant; HS highly significant; RNFL: retinal nerve fiber layer

Quadrant	Group 1A	Group 1B	Group 2	F/χ2*	p-Value
	Mild to moderate COPD	Severe to very severe COPD	Control		
Subfoveal choroidal thickness	248.40±8.94	176.85±7.43	254.40±8.94	1012.768	< 0.001**
Macular thickness	246.40±8.94	247.85±7.43	247.75±8.92	0.381	0.684
Peripapillary RNFL thickness					
Superior	131.40±8.94	124.45±11.51	126.40±8.94	5.142	0.007***
Inferior	125.48±9.26	117.40 ±9.06	129.48±9.26	17.460	< 0.001**
Nasal	85.18±6.49	84.30±4.98	90.18±6.49	10.774	< 0.001**
Temporal	84.98±7.12	76.85±7.43	87.98±7.12	24.780	< 0.001**
Average	106.76±4.39	100.75±4.66	108.51±4.39	32.166	< 0.001**

*Subfoveal Choroidal Thickness and* *Macular Thickness*

**Figure 1:**
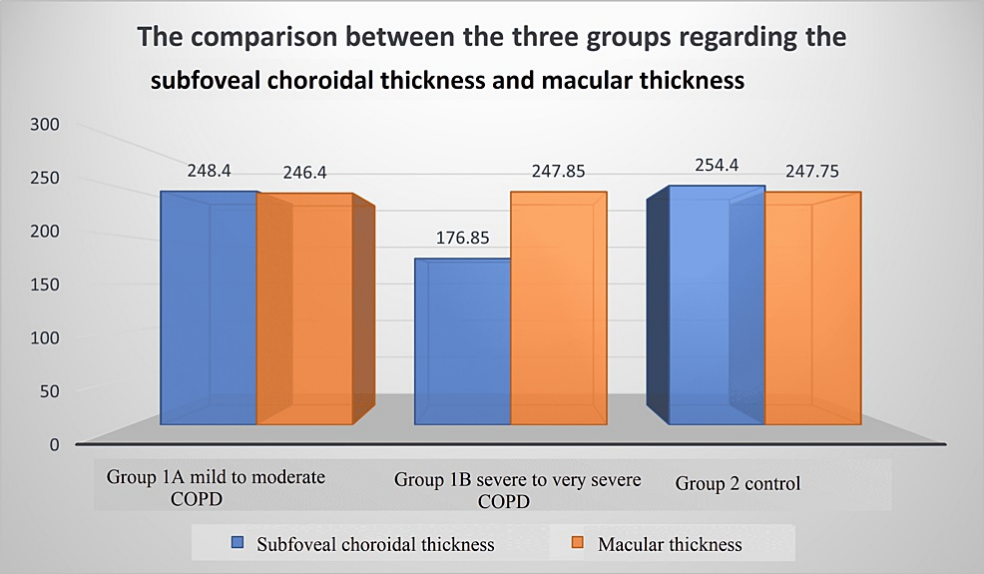
Comparison between the three groups regarding the subfoveal choroidal thickness and macular thickness COPD: chronic obstructive pulmonary disease

Subfoveal choroidal thickness (SFCT) was significantly thinner in the COPD groups compared to the control group. The thinnest SFCT was observed in the G1B group (176.85±7.43 μm) compared to the control group (254.40±8.94 μm) and G1A group (248.40±8.94 μm). These differences were statistically highly significant (p >0.001) (Figure 1). On comparing the macular thickness between the three groups there was no statistically significant difference (p=0.684) (Figure 1).

Peripapillary RNFL Thickness

**Figure 2:**
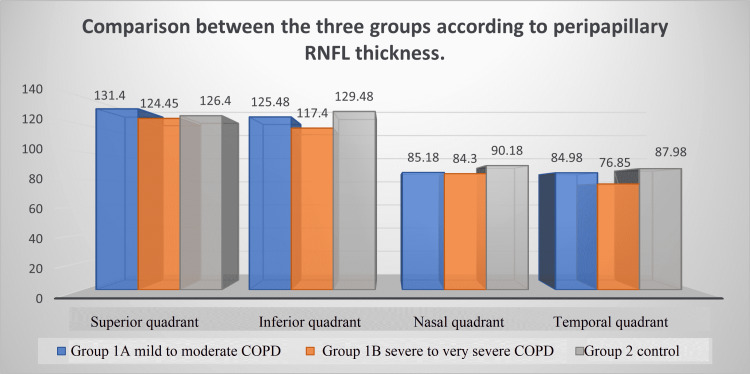
Bar chart showing comparison between the three groups according to peripapillary RNFL thickness RNFL: retinal nerve fiber layer; COPD: chronic obstructive pulmonary disease

Peripapillary RNFL was significantly thinner in the COPD groups than the control group in all quadrants (except the superior one) and in the average values (p <0.001). When comparing G1A and G1B, we found a statistically significant thinning in the RNFL in inferior and temporal quadrants in the G1B group (Figure 2).

Correlation between OCT measurements and pulmonary measurements of COPD groups

Subfoveal Choroidal Thickness

In the G1A group, there was a significant positive correlation between O_2_ saturation, forced expiratory volume in the first second (FEV 1), and forced expiratory volume in the first second/forced vital capacity (FEV1 /FVC) (Pearson’s correlation coefficients {r} of 0.321, 0.421, and 0.498 with a p-value of 0.001, 0.005, and 0.001, respectively).

Macular Thickness

In the G1A group, there was a significant positive correlation between MT and FEV 1 (Pearson’s correlation coefficients {r} of 0.119 with a p-value of 0.001). In the G1B group, there was a significant positive correlation between MT and FVC (Pearson’s correlation coefficients {r} of 0.277 with a p-value of 0.001); while there was a statistically significant negative correlation between the MT and FEV1 /FVC (Pearson’s correlation coefficients {r} of -0.126 with a p-value of 0.001).

## Discussion

Blood flow in the retina and choroidal blood is regulated by different mechanisms. It exhibits active autoregulation instead of autonomic innervation because of the absence of autonomic innervation. The autonomous nervous system and hormones are primarily responsible for regulating choroidal blood flow [9].

Our study found that all RNFL quadrants were thinner in COPD patients than in healthy controls. The RNFLs of patients with severe and very severe COPD are thinner in the inferior and temporal quadrants than those with mild to moderate COPD. Therefore, patients with severe COPD are likely to have thinner RNFL, indicating a correlation between the severity of the disease and RNFL loss. Subfoveal choroidal thickness (SFCT) was significantly thinner in the COPD groups compared to the control group. In this study, the measurements of macular thickness (MT) revealed no significant differences among the groups.

Similar findings were observed by Kocamış and Zorlu [10]. They reported a significant decrease in the SFCT in COPD patients during disease exacerbation and stability compared to the control group in their study. The choroid, being a highly vascular tissue can be influenced by variable systemic factors such as age, diabetes, and systemic blood pressure; thus, the significant reduction in SFCT in COPD patients could be related to the hypoxia and systemic inflammation present in the disease. No significant difference was found between the mean RNFL thicknesses of the COPD patients in the exacerbation and stable groups.

Ozcimen et al. found that the peripapillary CT was decreased in COPD patients when compared to controls, but the difference was significant only in the inferior segment [11]. Additionally, the average RNFL thickness in COPD patients was significantly reduced.

Gok et. al. found that all the quadrants of the RNFL of COPD patients were thinner than those of healthy individuals [7]. Nasal and average differences were significant. Additionally, those with severe COPD exhibited the greatest loss of RNFL, indicating a relationship (albeit not statistically significant) between the severity of the disease and the RNFL loss.

On the other hand, Ugurlu et al. found that the subfoveal CT was not significantly different between COPD patients and healthy persons, however, the inferior RNFL was significantly thinner in patients with COPD [12]. It is consistent with our findings.

There were some limitations to our work. As a result of the cross-sectional design, we cannot definitively state that COPD affects RNFL. As a result, some of the results of our study may have clinical applications. RNFL changes due to COPD may mimic the clinical findings in ocular diseases. Patients with COPD may lose RNFL due to vasoconstrictor production, impaired ocular blood flow, and retinal autoregulation, as well as increased vascular tone and resistance. A choroidal vascular network (which is controlled by autonomic nervous system) appears to be protected from the negative effects of COPD, providing nourishment to the outer layers of the retina. Finally, SD-OCT may be helpful in monitoring COPD progression.

## Conclusions

Hypoxia is indicated by an increase in PCO_2_ and a decrease in FEV1 / FVC. This cause RNFL and subfoveal choroidal thinning. This can be correlated with the severity of COPD. So, OCT findings can be used to check the microvascular hypoxia in COPD patients, before developing hypoxia/ischemia-induced retinal and disc edema.
